# A pan-African pathogen genomics data sharing platform to support disease outbreaks

**DOI:** 10.1038/s41591-023-02266-y

**Published:** 2023-05

**Authors:** Alan Christoffels, Gerald Mboowa, Peter van Heusden, Sello Makhubela, George Githinji, Sarah Mwangi, Harris Onywera, Ndodo Nnaemeka, Daniel Gyamfi Amoako, Idowu Olawoye, Amadou Diallo, Placide Mbala-Kingebeni, Samuel O. Oyola, Bright Adu, Christopher Mvelase, Pascale Ondoa, Fred Athanasius Dratibi, Abdourahmane Sow, Nicksy Gumede, Sofonias K. Tessema, Ahmed Ogwell Ouma, Yenew Kebede Tebeje

**Affiliations:** 1 Africa Centres for Disease Control and Prevention (Africa CDC), African Union Commission, Addis Ababa, Ethiopia.; 2 South African National Bioinformatics Institute, SAMRC Bioinformatics Unit, University of the Western Cape, Cape Town, South Africa.; 3 Nomazangwa Consulting, Haartbeesport, South Africa.; 4 KEMRI-Wellcome Trust Research Programme/KEMRI-CGMR-C, Kilifi, Kenya.; 5 Nigeria Centre for Disease Control, Abuja, Nigeria.; 6 National Institute for Communicable Diseases of the National Health Laboratory Service, Johannesburg, South Africa.; 7 College of Health Sciences, University of KwaZulu Natal, Durban, South Africa.; 8 African Centre of Excellence for Genomics of Infectious Diseases (ACEGID), Redeemer’s University, Ede, Osun State, Nigeria.; 9 Institut Pasteur of Dakar, Dakar, Senegal.; 10 Institut National de Recherche Biomédicale, Université de Kinshasa, Kinshasa, Democratic Republic of the Congo.; 11 International Livestock Research Institute (ILRI), Nairobi, Kenya.; 12 Noguchi Memorial Institute for Medical Research, College of Health Sciences, University of Ghana, Accra, Ghana.; 13 WHO Regional Office for Africa, Brazzaville, Republic of Congo.; 14 African Society for Laboratory Medicine (ASLM), Addis Ababa, Ethiopia.; 15 Regional Emergency Hub, WHO Regional Office for Africa, Nairobi, Kenya.; 16 West African Health Organization, Bobo-Dioulasso, Burkina Faso.

## Abstract

High-income countries have a wealth of genomics expertise that can be rapidly activated to deal with disease threats. African countries should invest in a federated data-management system for genomics epidemiology to deal with such threats better.

Infectious diseases pose a considerable threat to health in Africa — a threat that is compounded by an unpredictable rise in emerging and re-emerging infections, with more than 100 disease outbreaks reported annually within the continent^1^. In addition to the COVID-19 pandemic, Africa has grappled with three Ebola virus epidemics, a re-emergence of Marburg virus, continued antimicrobial resistance, and increased burden to health-care systems.

Effective control and monitoring of these disease threats have been successfully demonstrated in high-income countries, mainly in Europe and North America, in which next-generation sequencing (NGS) analysis of pathogens has been incorporated into disease surveillance systems, allowing for timely and in-depth pathogen characterization.

In high-income countries, prompt response to disease threats is facilitated by access to a critical mass of genomics and bioinformatics expertise, amassed over time and embedded in strong collaborative networks (Box 1). By contrast, fragmented surveillance systems in Africa required a multifaceted strategy to respond to COVID-19; notably, the mobilization of resources for 55 countries; the coordination of logistics to ensure timely deployment of equipment, supplies and reagents; and the provision of training in SARS-CoV-2 analytics to detect genetic variants and understand disease transmission.

The sustained use of genomics in Africa throughout the period 2020–2022 has been widely acknowledged. Its success can be ascribed to heightened coordination among regional bodies such as the Africa Centers for Disease Control and Prevention (Africa CDC), the World Health Organization Regional Office for Africa (WHO-AFRO), the West African Health Organization (WAHO) and others.

Local expertise was harnessed through a coordinated NGS pathogen surveillance network across Africa, supported by industry partners and funding agencies^2,3^. Unfortunately, the anticipated increase in NGS data has not been accompanied by adequate data management infrastructure within individual countries or across the continent. Nevertheless, the continental response to COVID-19 showed marked improvements in throughput, leading to reduced times to generate viral genome data^4^, which was facilitated by sustained regional-led capacity building, training and quality-assurance efforts. Evidence obtained from the African Pathogen Genomics Initiative, a project by the Africa CDC, identified data governance, including data sharing mechanisms, as a key element of a functional and real-time disease surveillance system in the continent.

## Ethical data sharing

Data sharing has been recognized as essential in a public health emergencies, underpinned by core principles such as ethics, timely response, equity, accessibility, transparency, fairness and quality^5^. Unlike other disease outbreaks, the COVID-19 pandemic saw an unprecedented sharing of viral genome data that has shaped global public health policies^6^. The success of this data-sharing strategy, including that observed in low- and middle-income countries (LMICs), can be attributed to increased access to NGS technology^2^ and perceived trust in international data repositories^7^.

However, researchers have raised concerns about a lack of equitable data-sharing protocols during the pandemic^7^. Recently, the ethics and data-sharing working group of the Public Health Alliance for Genomic Epidemiology (PHA4GE) outlined an ethical benefit-sharing framework for human genomics research^8^, addressing at a practical level many of these concerns. As such frameworks mature and become embedded in genomics initiatives, other elements of the data life cycle, such as data management and data storage, must also be interrogated.

Africa has a proven track record of leading international genomics projects that harness the rich genetic diversity of the continent^9^. However, these initiatives do not include a centralized African archive that can stand alongside global repositories. Although data cleaning is typically conducted locally, existing protocols prioritize the archiving of data to offshore facilities. The pervasive assumption is that there is no support for an African equivalent to the International Nucleotide Sequence Database Collaboration (INSDC). However, progress is being made in federated data-sharing approaches^10^.

During the COVID-19 pandemic, the Africa CDC established a technical working group to lead extensive continental stakeholder engagement meetings across 37 countries. The aim was to develop a vision for a data governance platform that can accelerate data sharing during disease outbreaks. Key factors were identified that can hinder, but also aid, efforts to incorporate genomics into disease surveillance across Africa, beyond COVID-19, with data governance a key unifying motif.

## Data infrastructure

Countries that have consistently invested in genomic technology and laboratory infrastructure demonstrated rapid national responses to COVID-19 (Box 1). With a few exceptions, this was not the case in Africa. A potentially game-changing pre-pandemic decision by the Africa CDC to strengthen NGS capacity across the continent was compromised by limited investment in data infrastructure. Putting this capacity in place requires a continent-wide vision that is adopted by local, regional, and international stakeholders to minimize duplication.

## Data protection

A close look at Africa’s personal data protection landscape shows that there has been a concerted effort among several countries to develop legal frameworks to protect personal data^11^. Nevertheless, evolving data protection policies across the continent creates uncertainty and no impetus for harmonization of policy frameworks across borders, or adherence. Establishing national pathogen surveillance policy frameworks can address these gaps.

## Data access

Data protection (security of data) must be disentangled from data privacy (data governance) and its associated administrative compliance processes. For transparent and trusted data storage, continental and global data management and sharing standards must be adopted by all players. The COVID-19 pandemic provided the impetus to explore solutions to these complex concerns and opened doors for suitable public–private partnerships.

## Laboratory surveillance systems

The Integrated Disease Surveillance and Response (IDSR) strategy aims to improve disease surveillance and the use of data for timely detection of and response to communicable and noncommunicable diseases in African countries. In line with the IDSR, Africa has a track record of quality surveillance data monitoring of disease outbreaks such as Ebola^12^, mpox^13^ and polio^14^. These programs would benefit from genomics technologies to deliver an even better public health service.

## Core digital platform capabilities

The continental drive for genomic surveillance of SARS-CoV-2 has re-ignited discussion on the need for a trusted African genome archive to strengthen the broader uptake of genomic technology and data governance in Africa. It is possible to imagine the INSDC as a federation across seven continents, with the genomic scientific community contributing their data to localized repositories that can interface with the rest of the world.

**Fig. 1 ∣:**
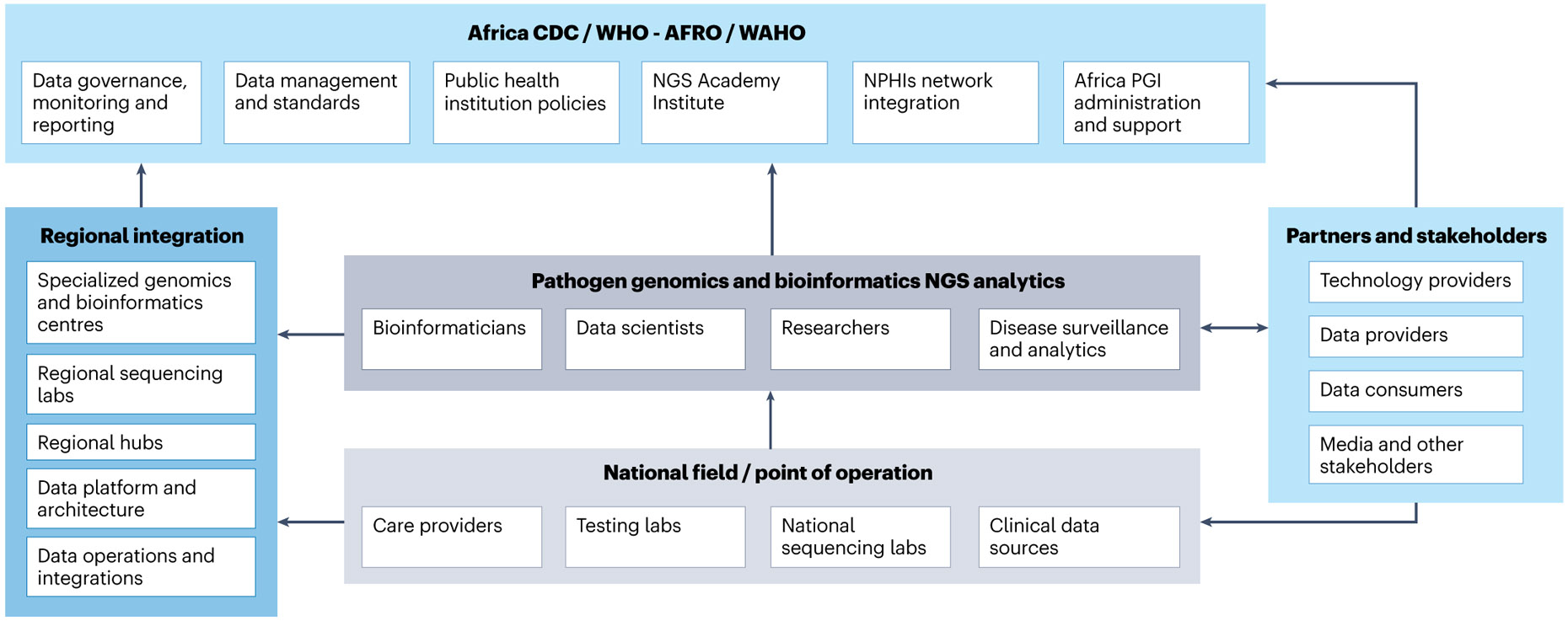
A pathogen genomics ecosystem for Africa. The complexity of the genomics landscape in Africa to support disease surveillance requires integrated data streams among public health practitioners, scientists, technology providers, government and health workers. NPHIs, National Public Health Institutes.

The ever-increasing data stream generated by NGS data analytics across Africa should support an ecosystem that informs data-driven decisions to prevent and respond to locally relevant diseases (Fig. 1). The complexity of the genomics landscape in Africa to support disease surveillance lies not only in managing volumes of data but in the ability to integrate data streams among a wide range of stakeholders.

A continental technology roadmap for a trusted data platform for routine pathogen data management in general and for immediate application to SARS-CoV-2 genome data was conceptualized through a series of stakeholder workshops during the past two years. These continental workshops gave rise to the Africa CDC-Africa Pathogen Genomics Initiative (PGI) data architecture technical working group (DTWG), who identified core digital platform capabilities as well as the associated management and support intrastructure that will realize the envisaged African pathogen genomic shared data platform (Table 1). The continental roadmap has started with an initial technical and stakeholder implementation of our data platform. It is being built in parallel with political and legal consultation to refine data-sharing agreements and support national data governance frameworks. The six pillars for such a platform would be: adoption and change management; program management; user experience; data products and data services; data management; and core infrastructure.

## Open-source technology platforms

Open-source technology platforms are key drivers of innovation and accelerate genomics discovery, as demonstrated by visualization tools^15^, workspace and application management systems^16-18^, data management platforms (https://www.overture.bio/documentation/) and others. There is an immediate need for a pilot data management platform to accommodate locally produced SARS-CoV-2, Ebola and Mpox genomic data, with built-in flexibility for other relevant pathogens. This platform is already being built to reduce turnaround time from sample collection to sharing, enable real-time genomic data utilization for public health decision making, and empower the overall pathogen genomic data sharing ecosystem across the continent.

## Federated data management

Federated data can be made available as objects using a standardized application programming interface (API), the ‘S3 API’. This API is implemented by commercial cloud vendors such as Amazon Web Services, Microsoft Azure and Google Cloud as well as software such as Ceph Object Gateway, OpenStack Object Storage and MinIO. These implementations enable data owners in public health and the wider scientific community to decide on the best architecture for their data storage, taking into account legal, policy and technical constraints. The shared data platform will be implemented on Amazon Web Services, Microsoft Azure and Google Cloud to future-proof the shared pathogen genomics archive and ensure that it does not suffer from vendor lock-in

## Pathogen analysis tools

A wide range of pathogen analytics tools^15,19-22^ is tailored for public health use, but few funding opportunities are available globally to develop methods or algorithms for the public health setting. Global initiatives such as the PHA4GE have attempted to break this implementation barrier by providing resources to catalyze collaborations in public health laboratories.

## Helpdesk support

Core support functions for genomics and bioinformatics queries are usually handled as requests on social media, via emails, bioinformatics-specific Slack communities, or helpdesks driven by the research community^23^. A pan-African pathogen genomics help-desk (https://africapgi.freshdesk.com) has already been established to leverage existing expertise within the African pathogen genomics ecosystem and beyond, and will evolve towards multi-lingual support.

## Data curation

Careful curation of data submitted to a shared data platform will be essential to ensure that researchers adhere to the FAIR principles of data management^24^. Partnering with the international biocuration community will help to develop and nurture a cohort of curators that can support an evolving genomics archive in LMICs.

## A global imperative

Data management and analytics to support data-driven decision making in public health is a global imperative, requiring continuous engagement with international disease surveillance stakeholders and technology platform developers. The response to future pandemics must be balanced against measures to tackle current disease outbreaks affecting the African continent, which place great burden on health-care systems. An African pathogen genomics shared data platform would seek to consistently implement capabilities, processes, resources, and systems for cost-effective NGS data analytics to respond to current disease outbreaks and potential future threats.

## Figures and Tables

**Table 1 ∣:** Pillars of an African pathogen genomics shared data platform for disease surveillance

Pillar	Description
Adoption and change management	Regional organizations including Africa CDC, WHO-AFRO and WAHO will drive development and implementation for policies, processes and system changes to enable adoption of a data platform to support pathogen genomics surveillance in Africa.
User experience	Standardized and consistent user-centric workflows, and value-driven processes to enable seamless and cost-effective data collection, sharing and use across the envisaged Africa pathogen genomic ecosystem.
Data services and products	Products or services that are consumable predominantly in a digital form and associated with the specific digital value such as data analytics, visuals and e-publications related to pathogen genomic information.
Data management	Consistent processes, practices, tools and controls for data acquisition, ingestion, integration, analysis, usage and decision making.
Program management	Coordination among key political stakeholders, partners, interest parties and the envisaged operational environment and networks of pathogen genomics and bioinformatics for optimized continent-wide capacity.
Core infrastructure	Technical platform composed of application and infrastructure components that can be rapidly reconfigured using agile methodologies, and cloud-native computing.

## BOX 1 Lessons learnt from rapid national responses to COVID-19

**Table:**

Strong research communities allowed countries to mobilize rapidly, for example COG-UK submitted a national proposal within 4 weeks.
Pathogen genomics communities have a history of contributing skills, analysis and infrastructure to other disease threats.
Onboarding of genomics technology in national public health laboratories took years to achieve.
Genome Canada’s rapid response capability was 20 years in the making through investment to support genomics research by academia and industry.
